# Draft Genome Sequence of Fungus *Clonostachys rosea* Strain YKD0085

**DOI:** 10.1128/genomeA.00538-16

**Published:** 2016-06-23

**Authors:** Shuai Liu, Yaowen Chang, Xujia Hu, Xuanyun Gong, Yingtong Di, Jinyan Dong, Xiaojiang Hao

**Affiliations:** a Key Laboratory of Eco-Environments in Three Gorges Reservoir Region of Ministry of Education, School of Life Science, Southwest University, Chongqing, China; b State Key Laboratory of Phytochemistry and Plant Resources in West China, Kunming Institute of Botany, Chinese Academy of Sciences, Kunming, Yunnan, China; c Shenyang Pharmaceutical University, Shenyang, Liaoning, China; d Kunming University of Science and Technology, Kunming, Yunnan, China

## Abstract

Here, we report the draft genome sequence of *Clonostachys rosea* (strain YKD0085). The functional annotation of *C. rosea* provides important information related to its ability to produce secondary metabolites. The genome sequence presented here builds the basis for further genome mining.

## GENOME ANNOUNCEMENT

The fungus *Clonostachys rosea* belongs to the family *Bionectriaceae* and was previously described as *Gliocladium roseum* (1). *C. rosea* is widely distributed all over the world. The destructive force of this fungus, as a biological control agent, is very strong in nematodes, insects, and many plant-pathogenic fungi, such as *Botrytis cinerea* and *Fusarium graminearum*. Except for the secretion of cell wall-degrading enzymes, *C. rosea* can produce a wide range of bioactive secondary metabolites (2–9).

Genomic DNA of *C. rosea* strain YKD0085 was obtained from a sample cultured in *Aspergillus* minimal medium (AMM) and then sequenced on an Illumina HiSeq 2000 platform. *De novo* assembly of the reads was performed using the SOAP*denovo* assembly protocol version 1.05 (10). DNA sequencing resulted in 1,504,639,412 raw reads, where 78,717,175 unique reads were obtained (estimated genome coverage, 31-fold) and used for genome assembly. The resulting assembly consists of 787 scaffolds and 55.18 Mb (*N*_50_, 528,196 bp; *N*_90_, 75,119 bp). The total G+C content was 49.46%.

Protein-coding gene models were predicted using *de novo* prediction tools Genscan (11), Augustus (12), and the homology-based gene prediction tool GeneWise (13), with the default parameters. The homology-based and *de novo* gene sets were merged to form a comprehensive and nonredundant reference gene set by Glean (14). The functional annotation of predicted gene models was mainly based on homology to known annotated genes, and BLAST was the tool mainly used in our analyses. We aligned all protein models by BLASTP to Swiss-Prot and NCBI nr and also mapped them onto functional terms, including GO (15) and KEGG pathways (16) (BLASTP cutoff e value. <1e - 7). The final structural gene prediction resulted in 16,120 gene models. By homology search, we mapped 10,006 predicted proteins to GO terms. The annotation revealed that the genome encodes 28 nonribosomal peptide synthetases, 39 polyketide synthases, and 15 terpenoid synthases. The *C. rosea* genome revealed a rich repertoire of secondary metabolite-encoding genes.

### Nucleotide sequence accession numbers.

The draft genome sequence has been deposited at GenBank under the accession no. LWRQ00000000.The version described in this paper is the first version, LWRQ01000000.
